# Clinical Application of Liquid Biopsy in Targeted Therapy of Metastatic Colorectal Cancer

**DOI:** 10.1155/2017/6139634

**Published:** 2017-01-23

**Authors:** Jörg Trojan, Susanne Klein-Scory, Christine Koch, Wolff Schmiegel, Alexander Baraniskin

**Affiliations:** ^1^Gastroenterology, Department of Medicine, Goethe University Frankfurt, Frankfurt, Germany; ^2^IMBL Medical Clinic, Universitätsklinikum Knappschaftskrankenhaus Bochum, Bochum, Germany; ^3^Department of Medicine, Hematology, and Oncology, Ruhr-University of Bochum, Bochum, Germany

## Abstract

*Background.* Colorectal cancers (CRC) shed DNA into blood circulation. There is growing evidence that the analysis of circulating tumor DNA can be effectively used for monitoring of disease, to track tumor heterogeneity and to evaluate response to treatment.* Case Presentation.* Here, we describe two cases of patients with advanced CRC. The first case is about a patient with no available tissue for analysis of RAS mutation status. Liquid biopsy revealed RAS-wild-type and the therapy with anti-EGFR (epidermal growth factor receptor) monoclonal antibody cetuximab could be initiated. In the second case, the mutational profile of a patient with initial wild-type RAS-status was continually tracked during the course of treatment. An acquired KRAS exon 3 mutation was detected. The number of KRAS mutated fragments decreased continuously after the discontinuation of the therapy with EGFR-specific antibodies.* Conclusion*. Liquid biopsy provides a rapid genotype result, which accurately reproduces the current mutation status of tumor tissue. Furthermore, liquid biopsy enables close monitoring of the onset of secondary resistance to anti-EGFR therapy.

## 1. Introduction

Colorectal cancer (CRC) is one of the most prevalent cancers and a leading cause of cancer mortality worldwide. The anti-epidermal growth factor receptor (anti-EGFR) monoclonal antibodies cetuximab and panitumumab have improved clinical outcomes for patients with all wild-type RAS metastatic colorectal cancer (mCRC) and are part of standard first-line chemotherapy for these patients, in particular for patients with left-sided mCRC. The exclusion of RAS mutations prior to therapy is mandatory for application of anti-EGFR antibodies. The current gold standard is the determination of RAS status in surgical or bioptic specimens.

Recently, liquid biopsy, a noninvasive blood-based RAS mutation analysis of cell-free circulating tumor DNA (ctDNA), has been reported as a suitable method for mutational analysis in patients with mCRC [1, 2]. In contrast to tissue based RAS status determination, liquid biopsy is able to address tumor heterogeneity and treatment-related dynamic changes of molecular profiles [3–6]. The possibility to monitor the RAS status by liquid biopsy [2–6] during disease progression directed the focus onto the dynamic of mutational changes during therapy. The benefit of therapy adaptation for patients based on the monitored RAS status is not addressed so far by studies concerning mCRC.

In two case reports, we present the significance of liquid biopsy for the therapy of mCRC.

**Figure 1:**
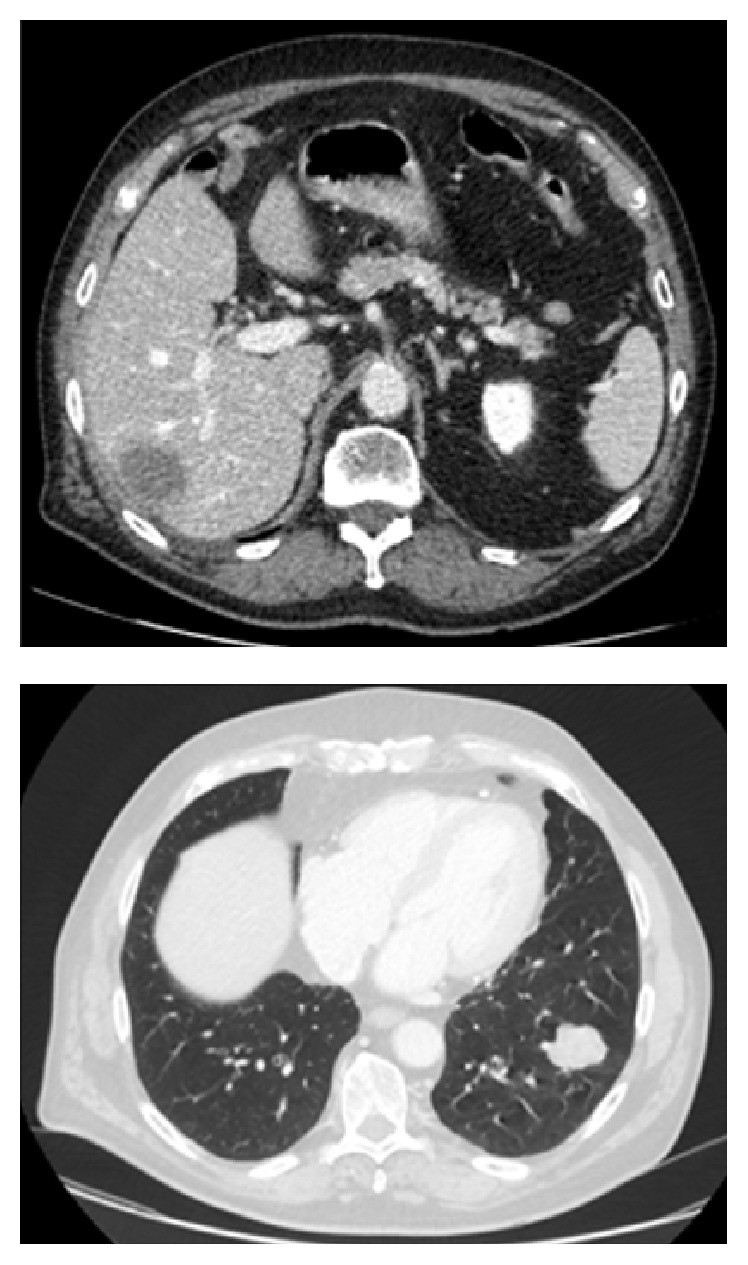
Computed tomography scan of the patient of case 1 at the diagnosis of metachronous hepatic and pulmonary metastases.

*Case  1*. A 74-year-old man was diagnosed with a right-sided adenocarcinoma (ascending colon) in 2006 and subsequently underwent a right hemicolectomy. Histopathology revealed a UICC stage II (pT3, N0 (0/16), L0, M0 and R0), and, accordingly, no adjuvant therapy was given. In 2014, he presented again with hepatic and pulmonary metastases (Figure 1). The relapse of colorectal cancer was confirmed by a CT-guided biopsy of a hepatic metastasis. The interdisciplinary tumor board recommended the initiation of palliative chemotherapy dependent on RAS mutation status. Unfortunately, there was not sufficient malignant tissue in the biopsy to define the RAS mutation status. Besides this, the tissue from the hemicolectomy was not available anymore. Under these circumstances, a palliative therapy with bevacizumab combined with capecitabine was started. After the initial response, the disease was progressive. The patient was in good general health (ECOG 0) and refused further biopsy; therefore, a liquid biopsy as previously reported was performed [1, 2]. No RAS mutations were detected and a second-line therapy including cetuximab was initiated.

**Figure 2:**
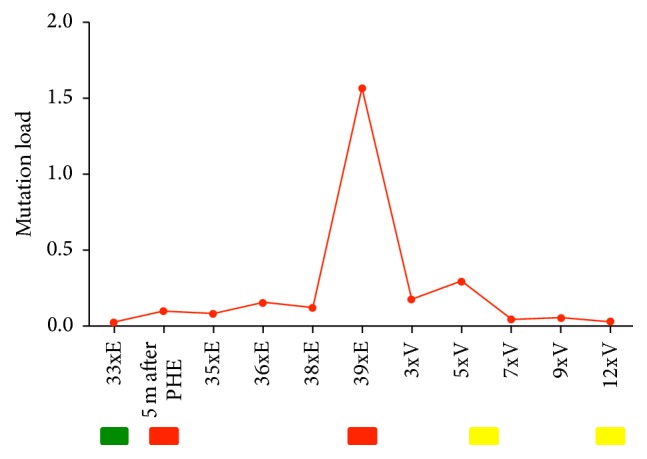
Dynamics of a KRAS mutant clone in plasma samples of the patient in case 2. The red line indicates the frequency of KRAS exon 3 mutation (percentage of alleles) detected in circulating DNA at the indicated time points. Progressive disease occurred after the long-term treatment with anti-EGFR antibodies. After interruption of anti-EGFR therapy; the KRAS mutation load distinctly declined and remained below the limit of detection across subsequent lines of treatment. “E” represents therapy with anti-EGFR antibodies and chemotherapy and “V” therapy with anti-VEGF antibodies and chemotherapy; “PHE” acts for partial hepatectomy and “m” for month. Results of follow-up are indicated below the graphs: green box, partial remission; yellow box, stable disease; and red box, progressive disease.

*Case  2*. A 37-year-old woman was diagnosed with a rectal adenocarcinoma with synchronous dominant hepatic and few pulmonary metastases in 2013. The molecular genetic examination of tumor tissue revealed a RAS and BRAF wild-type. Microsatellite instability was also ruled out. After the deep anterior rectum resection, a systemic chemotherapy combined with cetuximab was initiated, and after confirmation of a partial response this treatment was continued for 33 cycles. During this period, no RAS mutation was detectable by liquid biopsy (Figure 2) [1, 2]. However, after the 33rd cycle (July 2015) of treatment with continuous application of cetuximab, low frequency KRAS exon 3 mutations were detectable by liquid biopsy in the plasma of the patient for the first time. Due to a deep tumor response, a partial hepatectomy was performed and chemotherapy was paused. Four months later, in November 2015, progression occurred and the therapy was reinitiated. After four additional cycles, the low frequency KRAS exon 3 mutations, which have been noted before, were detectable by liquid biopsy again. During the following two cycles, the KRAS exon 3 mutation load increased more than tenfold. At that time, a follow-up imaging revealed progression of the hepatic metastases. Thus, the therapy was changed to aflibercept, an inhibitor of VEGF, in combination with chemotherapy. During this further treatment, the number of KRAS mutated fragments declined continuously and remained below the limit of detection. The follow-up imaging after 6 and 12 cycles revealed a stable disease. CEA and CA19-9 were not elevated throughout the evaluation period.

## 2. Discussion

Plasma RAS testing in blood was performed by liquid biopsy using BEAMing (Sysmex Inostics, Hamburg, Germany), allowing the evaluation of 34 mutations in KRAS and NRAS of exons 2, 3, and 4. BEAMing RAS analysis demonstrated a high concordance of plasma and tissue results of 92.2% [1, 2].

Liquid biopsy has the potential to complement tumor tissue genotyping. In case of lacking availability of tissue; liquid biopsy is a new option to determine the mutation status as shown in case 1 and thereby to allow patients to benefit from further lines of treatment using an anti-EGFR-targeted agent.

It has been recently shown that, due to the intrinsic molecular heterogeneity of cancer, the RAS mutation status of primary tumors and metastases differs in about 20% [7–10]. The dynamical adaption of the CRC genome towards application of chemotherapeutics may also be taken into account and especially targeted drugs like anti-EGFR-antibodies leading to acquired (secondary) resistance [3, 4]. To monitor this therapy-relevant phenomenon of clonal selection, the mutational profiles of patients with CRC should be continually tracked during the course of treatment as we did in case 2. Here, we followed a resistant clone in the blood of a patient who initially benefitted from an anti-EGFR-antibody for more than 2 years and then experienced disease progression shortly after the mutation load increased. This indicates that alterations in plasma DNA mutant fragments as a result of a clonal selection are a surrogate marker for disease progression. The mutation load declined when EGFR-specific antibodies were withdrawn. These findings fit into the common pattern that was previously described which shows that mutant allele levels gradually decrease after the discontinuation of the therapy with EGFR-specific antibodies. Furthermore, our results are in accordance with the recently published observations of Toledo et al., demonstrating that reintroduction of chemotherapy may block the growth of the resistant clone and regain control of the disease in case of a moderate rise of mutation load. Thus, the level of increase of mutation load seems to predict the reversibility of resistance [6]. This knowledge opens up new perspectives for the anti-EGFR rechallenge [3, 4].

Rechallenge with EGFR antibodies was recently reported to be effective and generated responses and long-lasting disease control [3, 4, 11]. Accordingly, liquid biopsy plays a crucial role in determination of the appropriate point of time for initiation of the rechallenge therapy.

In conclusion, the analysis of ctDNA, also known as liquid biopsy, delivers a rapid genotype result, which precisely reproduces the mutation status of tumor tissue and may complement or in some cases even substitute tumor tissue genotyping [1, 2]. The sampling bias caused by tissue heterogeneity may be overcome by the liquid biopsy and may enable a more personalized treatment in particular with regard to targeted therapies. Moreover, liquid biopsy can be used to dynamically monitor the onset of secondary resistance to anti-EGFR therapy through the selection of further molecular alterations and provide essential support for choosing the subsequent therapy.
